# Systemic NF-κB-mediated inflammation promotes an aging phenotype in skeletal stem/progenitor cells

**DOI:** 10.18632/aging.203083

**Published:** 2021-05-25

**Authors:** Anne Marie Josephson, Kevin Leclerc, Lindsey H. Remark, Emma Muiños Lopeź, Philipp Leucht

**Affiliations:** 1 NYU Grossman School of Medicine, NYU Langone Orthopedics, New York, NY 10016, USA; 2 NYU Grossman School of Medicine, Department of Cell Biology, New York, NY 10016, USA

**Keywords:** regeneration, skeletal stem cell, aging, inflammation, nuclear factor kappa B

## Abstract

Aging tissues undergo a progressive decline in regenerative potential. This decline in regenerative responsiveness has been attributed to changes in tissue-specific stem cells and their niches. In bone, aged skeletal stem/progenitor cell dysfunction is characterized by decreased frequency and impaired osteogenic differentiation potential. This aging phenotype ultimately results in compromised regenerative responsiveness to injury. The age-associated increase of inflammatory mediators, known as inflamm-aging, has been identified as the main culprit driving skeletal stem cell dysfunction.

Here, we utilized a mouse model of parabiosis to decouple aging from inflammation. Using the Nfkb1^-/-^ mouse as a model of inflamm-aging, we demonstrate that a shared systemic circulation between a wild-type and Nfkb1^-/-^ mouse results in an aging phenotype of the wild-type skeletal stem and progenitor cells, shown by CFU-fs and osteogenic and adipogenic differentiation assays. Our findings demonstrate that exposure to an inflammatory secretome results in a phenotype similar to the one observed in aging.

## INTRODUCTION

Bone fractures are the most common large-organ, traumatic injuries in humans; however our capacity to heal and form new bone in response to injury diminishes as we age [1, 2]. When a young, healthy adult fractures a bone, skeletal stem and progenitor cells (SSPCs) proliferate and differentiate into osteoblasts. This process ultimately results in complete regeneration of the skeletal element with its biomechanical properties. In the elderly, however, this process is compromised and often results in an impaired healing response [3, 4].

A well-balanced inflammatory response is crucial for skeletal repair after trauma [1]. In aging individuals, an imbalance between adaptive and innate immunity, as well as a progressive accumulation of senescent cells lead to elevated levels of pro-inflammatory mediators in tissues, a process known as ‘inflamm-aging’ [5, 6]. This chronic unbalanced elevation of pro-inflammatory cytokines has been shown to inhibit regeneration in a variety of tissues [7]. Our previous work identified inflamm-aging as the main culprit driving the decline in SSPC function, characterized by decreased SSPC number, decreased osteogenesis and increased adipogenesis [8]. Further, we observed that suppression of systemic inflammation led to a functional rejuvenation of the aged SSPC with increased SSPC number and improved osteogenic differentiation potential [8]. This work demonstrates that the systemic factors in the extrinsic environment of the SSPC greatly affect its function and differentiation capacity. We further questioned whether young, healthy SSPCs could be made “aged” by exposure to elevated inflammatory mediators. The *Nfkb1^-/-^* mouse has served as a model organism for low-level chronic inflammation in many studies [9–11]. The *Nfkb1^-/-^* mouse lacks expression of the p105 and p50 NF-κB proteins. Deletion of these two NF-κB subunits results in the inability to form p50:p50 homodimers (repressor of pro-inflammatory gene expression), while still being able to generate RelA-containing NF-κB dimers (activators of pro- inflammatory gene expression). As a consequence, NF-κB1^-/-^ mice display increased levels of sterile chronic inflammation. Here, we utilized an experimental system in which young, healthy SSPCs are exposed to a drastically heightened pro-inflammatory environment *in vivo*. Parabiotic pairings were established between *Nfkb1^-/-^*; *Beta-actin:GFP* and wild-type (C57BL/6) mice (WT). In this model, young wild-type mice are surgically anastomosed to a *Nfkb1^-/-^*; *Beta-actin:GFP* mouse and will share circulation after two weeks [12]. We hypothesized that systemic pro-inflammatory factors in the *Nfkb1^-/-^* will impair skeletal stem cell function.

## RESULTS

**Figure 1:**
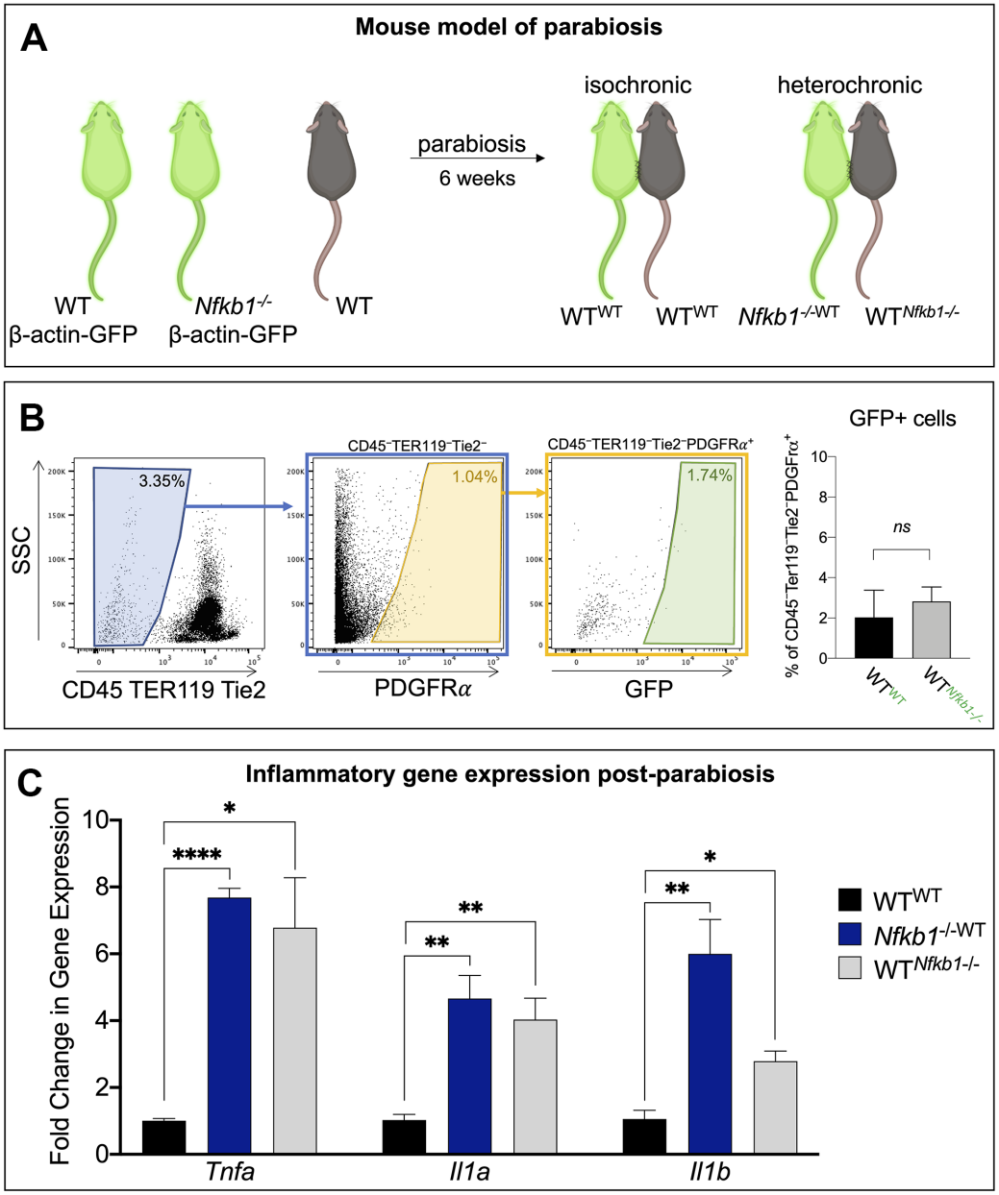
**Exposure to *Nfkb1^-/-^* circulation results in increased inflammation in bone marrow compartment.** (**A**) Schematic representation of the parabiosis model in which one mouse shares circulation with another surgically-anatomized mouse. A wild-type mouse parabiosed to another wild-type mouse serves as the control (isochronic pair) and *Nfkb1^-/-^* mouse parabiosed to a wild-type mouse serves as the experimental group (heterochronic pair). Parabionts are noted as such wild-type parabiosed to wild-type, WT^WT^; *Nfkb1^-/-^* mouse parabiosed to wild-type, *Nfkb1^-/-^*^WT^; and wild-type parabiosed to *Nfkb1^-/-^* mouse, WT*^Nfkb1-/-^*. Green mice depict *Beta-actin* GFP mice (C57BL/6-Tg(CAG-EGFP)1Osb/J), brown mice represent wild-type mice. (**B**) Gating strategy demonstrating that parabiosis led to an insignificant transfer of SSPCs from one animal to the other. (**C**) After six weeks of shared circulation the bone marrow compartment of WT*^Nfkb1-/-^* mice displayed higher expression of the inflammatory mediators *Tnfa, Il1a* and *Il1b* compared to WT^WT^ controls. Conversely, exposure of WT circulation to the *Nfkb1^-/-^* bone marrow did not result in a reduced inflammatory status. (n=3, ns, non-significant, *P < 0.05, **P ≤ 0.01, *** P ≤ 0.001, **** P ≤ *0.0001).*

The use of GFP-transgenic mice allowed confirmation of a shared circulatory system two weeks post-parabiosis surgeries. Shared circulation results in the exposure of young WT SSPCs to an increased pro-inflammatory environment from the *Nfkb1^-/-^* mouse [12, 13]. We denote the mice as such, isochronic control pair: wild-type parabiosed to wild-type (WT^WT^); and heterochronic pair: *Nfkb1^-/-^* mouse parabiosed to wild-type (*Nfkb1^-/-^*^WT^) and wild-type parabiosed to *Nfkb1^-/-^* mouse (WT*^Nfkb1-/-^*) (Figure 1A). The use of β-*actin:GFP* transgenic mice as one member of a pair also allowed us to distinguish the SSPCs from each animal participating in stem cell function assays. SSPCs are conventionally defined by a suite of cell surface markers that include PDGFRα or CD51 [14, 15]. When SSPCs were isolated from the tibiae of isochronic and heterochronic parabiosed mice, flow cytometry revealed a very limited amount of transfer of GFP^+^PDGFRα^+^ SSPCs (< 3%) from the WT or Nfkb1^-/-^ mice (Figure 1B); this is consistent with other comparable parabiosis studies measuring the contribution of circulating, tissue-specific stem cells to the nontransgenic stem cell pool [12, 13]. This therefore indicates that any experimental effect on normal stem cell function is primarily due to the shared circulatory/inflammatory environment of the mutant parabiont, and not due to an extensive contamination of mutant cells.

We have previously shown that the majority of cultured bone marrow cells express the stem and progenitor markers PDGFRα and CD51, thus making them a suitable *in vitro* model system to study stem cell function [8]. After six weeks of shared circulation, the parabionts were sacrificed and we examined the functional consequence of exposure to a heightened NF-κB-mediated pro-inflammatory environment on WT SSPCs. We first examined whether a shared circulatory system resulted in an increased inflammatory gene expression within the local bone marrow compartment. Gene expression analyses for the inflammatory mediators tumor necrosis factor alpha (*Tnfa),* interleukin 1 alpha (*Il1a)* and interleukin 1 beta (*Il1b*) revealed significantly elevated expression in the wild-type parabiosed to *Nfkb1^-/-^* mouse (WT*^Nfkb1-/-^*) compared to isochronic controls (WT^WT^), although expression levels were still lower than in the *Nfkb1^-/-^* mouse parabiosed to wild-type (*Nfkb1^-/-^*^WT^) (Figure 1C). These inflammatory mediators are not only upstream of NF-κB pathway activation, but their expression is also regulated by NF-κB signaling [16, 17]. Additionally, increased expression of *Tnfa, Il1a* and *ll1b* is in line with the inflamm-aging phenotype observed in aging mice [16, 18].

**Figure 2:**
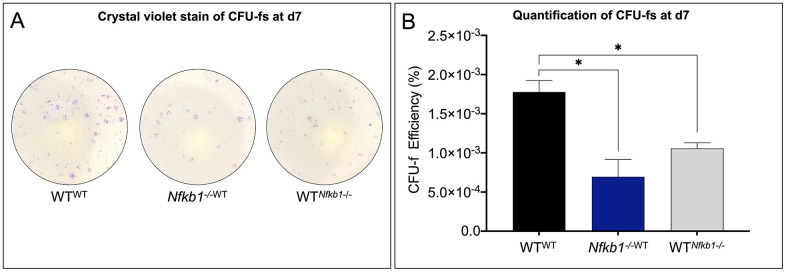
**SSPC frequency declines in response to increased NF-κB-mediated inflammation.** (**A**) SSPCs isolated from parabiosed animals six weeks post-surgery revealed that exposure to increased inflammation results in decreased SSPC number as revealed by colony forming unit (CFU) assays. (**B**) *Nfkb1^-/-^*^WT^ and WT*^Nfkb1-/-^* SSPCs gave rise to significantly fewer colonies than those isolated from the WT^WT^ parabionts as revealed by CFU-f efficiency (n=3, *P < 0.05).

We have previously demonstrated that inflamm-aging is associated with a significant decrease in SSPC number [8]. Therefore, we examined SSPC frequency using a colony forming unit (CFU) assay. Crystal violet stain to identify stem cell colonies demonstrated a significant decrease in CFUs in the experimental heterochronic groups compared to the isochronic control. Additionally, a significantly decreased number of SSPCs was observed in the experimental parabiont, WT*^Nfkb1-/-^*, compared to the control, WT^WT^ (Figure 2A). The fewest number of progenitors were observed in the *Nfkb1^-/-^*^WT^. Quantification of CFU efficiency (counted CFUs/cells originally seeded*100) confirmed these observations (Figure 2B). Having established an effect on SSPC frequency, we next set out to assess the effect of NF-κB-mediated inflammation on differentiation potential.

**Figure 3:**
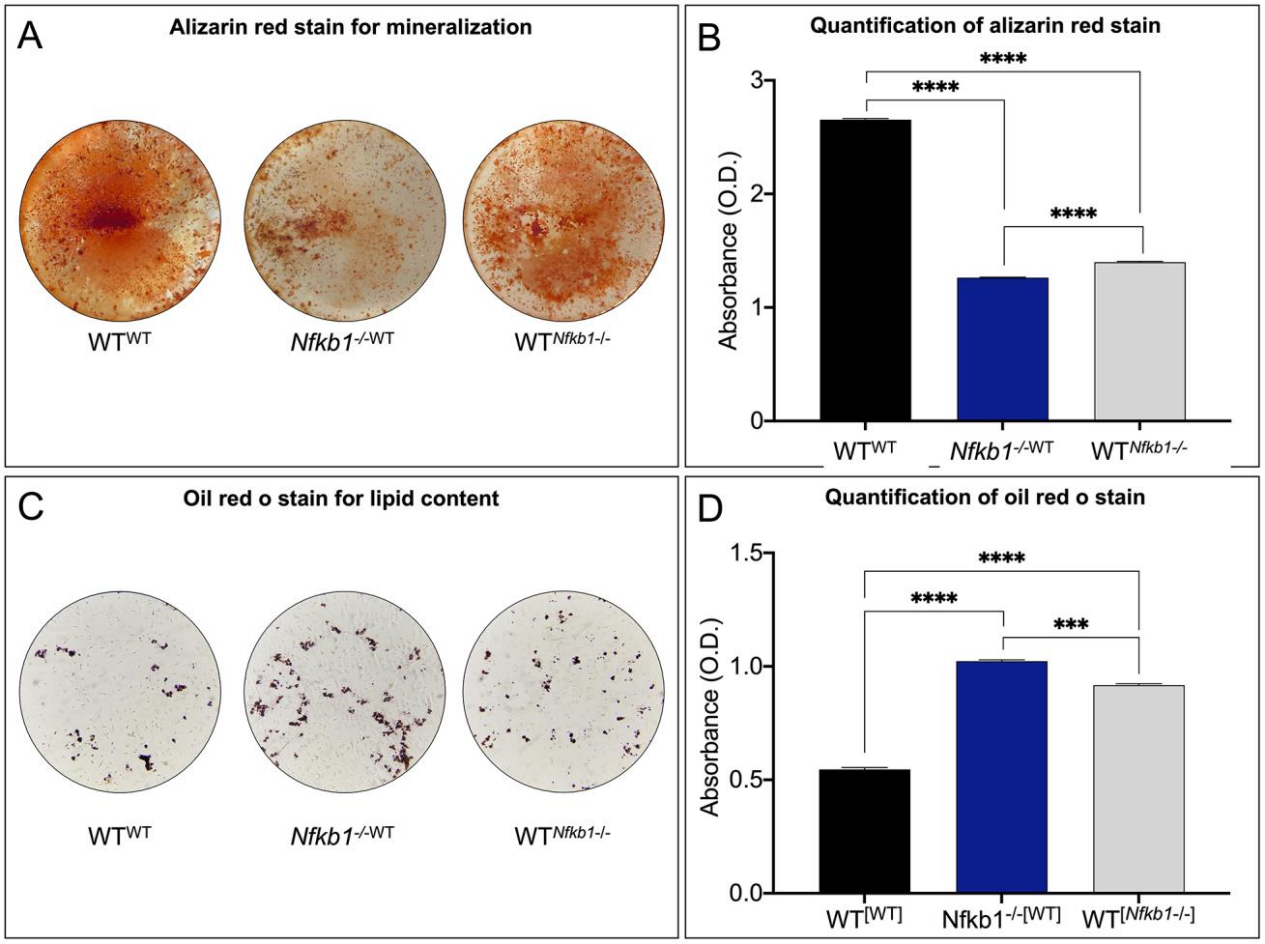
**Increased NF-κB-mediated inflammation leads to impaired differentiation potential.** (**A**, **B**) Osteogenic potential was assessed by Alizarin red staining (**A**), which demonstrated significantly reduced mineralization in *Nfkb1^-/-^*^WT^ and WT*^Nfkb1-/-^* samples compared to control (**B**) (n=3, **** P < 0.0001). (**C**, **D**) Significantly increased lipid accumulation was observed in *Nfkb1^-/-^*^WT^ and WT*^Nfkb1-/-^* samples as revealed by Oil Red O staining (**C**) and quantification (**D**) (n=3, *** P ≤ *0.001,* **** P ≤ *0.0001*).

Aging has been associated with a loss of osteogenesis and increased fatty degeneration within the bone marrow compartment [19]. To directly test whether inflammation can be attributed to this phenotype, we assayed the differentiation potential of the parabionts. Alizarin red staining was used to visualize the mineralized matrix after 14 days of culture in osteogenic medium. Staining (Figure 3A) and quantification (Figure 3B) revealed significantly decreased mineralization in *Nfkb1^-/-^*^WT^ and WT*^Nfkb1-/-^* compared to SSPCs isolated from the control, WT^WT^, suggesting that exposure to an increased pro-inflammatory environment impairs the osteogenic capacity of SSPCs. In line with the observed decrease in osteogenesis, oil red o staining for lipid droplets (Figure 3C) revealed that SSPCs isolated from *Nfkb1^-/-^*^WT^ and WT*^Nfkb1-/-^* mice exhibited increased adipogenic potential compared to the control. Quantification of oil red o staining was in line with these observations (Figure 3D). Collectively, these data confirmed that heightened NF-κB-mediated inflammation contributes to a decline in SSPC number and impaired differentiation potential.

## DISCUSSION

Aging is associated with increased skeletal stem cell dysfunction. We have previously shown that in humans, aging is accompanied by a large reduction in SSPC frequency and there is a direct correlation between stem cell number and time to bone fracture union [8]. Additionally, aged animals exhibit significant delays and deficiencies in osteogenesis following fractures [2, 20, 21] and this decrease in osteogenicity normally coincides with an increase in adipogenic capacity and bone marrow adiposity [22, 23].

Altogether, this aging SSPC phenotype of reduced frequency, decreased osteogenic potential and increased adipogenic potential results in compromised regenerative responsiveness to injury [8].

Crucially, our prior work has underpinned the increase inflammatory mediators associated with age, or inflamm-aging, as the driver behind this age-associated SSPC dysfunction [8], suggesting that the increase in inflammatory mediators in the environment of the SSPC negatively affects its function. In this work, we directly tested this hypothesis by utilizing a mouse model of parabiosis to assess the effect of NF-κB-mediated inflammation on young, healthy, wild-type SSPCs. In this approach, there is a very limited transfer of cells from one parabiont to another, ultimately signifying that exposure to elevated inflammatory mediators in the extracellular environment can have a potent effect on SSPC behavior, causing young SSPCs to take on an aged phenotype. These data demonstrate the extent to which extrinsic factors can intrinsically affect cells.

One might expect changes in skeletal stem cell behavior to affect bone homeostasis and a proper response to injury. Skeletal elements from either parabiont pair did not exhibit any gross morphological differences from WT mice and this in line with our previous work showing no differences in the skeletal architecture using microCT. However, a longer exposure to elevated levels of inflammation may be necessary to observe significant alterations in bone homeostasis due to impaired SSPC function. The stress of anastomosis precluded our ability to directly assess the function of SSPCs in an *in vivo* injury model, but the functional experiments in this study together demonstrate a significant defect in SSPC behavior. Indeed, previous research has shown that an inflammation-associated decline in normal SSPC function is associated with a decrease in SSPC engraftment potential following transplantation [8].

NF-κB is the fundamental transcriptional regulator of inflammation. Although we cannot rule out any secondary, noncanonical effects of the *Nfkb1* deletion, previous reports from our group and others have shown that *Nfkb1^-/-^*-mediated inflammation provokes similar phenotypes to that of middle-aged mice, including lower skeletal stem cell frequency, decreased and defective osteogenesis, and increased adiposity, among others. We also demonstrated that anti-inflammatory treatment with sodium-salicylate could reverse these phenotypes and, in essence, rejuvenate the stem cell pool, supporting the notion that inflammation arising from the deletion of *Nfkb1* is the main culprit of deficient SSPC function.

Our experiments suggest that systemic factors from NF-κB-mediated inflammation can modulate the molecular signaling pathways critical to osteogenesis and adipogenesis. Inhibition of osteogenesis and activation of adipogenesis could result from dysregulation of Jak/Stat signaling. The Jak/Stat signaling pathway has been implicated as a critical effector of inflammation and is activated by inflammatory cytokines [24]. Recent work has also pointed to Jak/Stat signaling as a critical effector in reciprocal regulation of adipo- and osteogenesis. In SSPCs, Jak/Stat signaling inhibited osteogenesis via the Wnt pathway and promoted adipogenesis by upregulation of CCAAT-enhancer-binding protein alpha, C/EBPɑ [25]. In addition to regulation of the Wnt pathway by Jak/Stat, NF-κB has been shown to suppress Wnt signaling, resulting in decreased osteogenesis through suppression of beta-catenin [26]. As it has been proposed that default differentiation of the SSPC is the adipocyte [23], our results suggest that NF-κB-mediated inflammation promotes adipogenesis at the expense of osteogenesis.

The systemic environment of a young, wild-type progenitor is one that promotes successful SSPC function and regeneration, whereas that of an aged animal fails to support proper SSPC function. Previous work in bone [8, 27] and other tissues, such as skin [28], has suggested that age-related alterations in the inflammatory milieu underlie regeneration deficiencies. These observations are in line with evidence suggesting that progenitor cells preserve much of their intrinsic potential even when aged, but that age-related changes in the systemic environment inhibit activation of cellular programs that are necessary for proper tissue regeneration [12]. Correspondingly, our work demonstrates that despite the intrinsic capacities of young, healthy SSPCs, their function is largely affected by their environment. As such, systemic NF-κB-mediated inflammation caused young SSPCs to take on an aging phenotype. This work provides valuable insight into triggers that cause age-associated SSPC dysfunction. As NF-κB is a known transcriptional effector the mediates chronic inflammation in aged skin, liver, muscle and nervous system, this work has the potential to provide a therapeutic insight to improve the regenerative capacity of stem cells in a variety of tissues beyond just bone [29, 30].

## MATERIALS AND METHODS

### Animals

All procedures were approved by the New York University Committee on Animal Research and were performed according to institutional guidelines and regulations. C57BL/6 mice, *Nfkb1* knockout mice (B6; 129P-*Nfkb1^tmBal^*/J, Jax no. 002849), *Beta-actin* GFP mice (C57BL/6-Tg(CAG-EGFP)1Osb/J, Jax no. 003291) were purchased from Jackson Laboratory (Bar Harbor, ME) and bred in the mouse facility at the New York University Robert I. Grossman School of Medicine. Mice were aged until 3 months of age. Mice of both male and female sex were randomly assigned to the experimental groups.

### Parabiosis

Parabiosis was performed as previously published [13]. Briefly, after anesthesia the lateral sides of each mouse was shaved, and skin incisions were made from the olecranon to the knee joint of each mouse. A single 2-0 silk suture and tie was used to attach the olecranon and knee joints and the dorsal and ventral skins were approximated by continuous suture. All procedures were approved by the New York University Robert I. Grossman School of Medicine’s Committee on Animal Research. Animals were euthanized six weeks after surgery.

### Isolation and culture of SSPCs

Tibial and femoral SSPCs were isolated by centrifugation for *in vitro* experiments [31]. Bone marrow was resuspended in growth media (GM) [DMEM containing 10% FBS and 1% penicillin/streptomycin; ThermoFisher Scientific] and then plated in 150cm^2^ tissue-culture flasks. Media changed the following day and then every two days. All cellular assays were performed with SSPCs at passage one from at least three different mice in technical replicates.

### RNA isolation and quantitative real time-PCR (qRT-PCR)

RNA was isolated as per manufacturer’s instructions using the RNeasy Plus Mini Kit (Qiagen), and reverse-transcribed using the iScript cDNA Synthesis Kit (BioRad). The cDNA was amplified for specific targets using specific primers (Table 1) and RT^2^ SYBR Green ROX PCR Master Mix (Qiagen) in a QuantStudio3 Real-Time PCR System. Results are presented as 2^–ΔΔCt^ values normalized to the expression of 18S and young or negative control samples. Means and SEMs were calculated in GraphPad Prism software.

**Table 1:** PCR primers.

**Primer Name**	**Sequence (5’ – 3’)**
*18s* FOR	ACGAGACTCTGGCA TGCT AACT AGT
*18s* REV	CGCCACTTGTCCCTCTAAGAA
*Il1a* FOR	GACGTTTCAGAGGTTCTCAGAG
*Il1a* REV	CCATGAGCGCATCGCAATC
*Il1b* FOR	GCAACTGTTCCTGAACTCAACT
*Il1b* REV	A TCTTTTGGGGTCCGTCAACT
*Tnfa* FOR	CTGTAGCCCACGTCGTAGC
*Tnfa* REV	TTGAGATCCATGCCGTTG

All primers were purchased from Integrated DNA Technologies.

### Colony forming unit (CFU) assay

5×10^5^ cells/cm^2^ were seeded immediately post isolation onto 24-well plates in GM. On day 7, cells were stained with 1% crystal violet in methanol. Stained colonies of >20 cells were scored as a CFU-f. CFU efficiency was calculated (counted CFUs/cells originally seeded*100). Means and SEMs were calculated in GraphPad Prism software.

### Osteogenic differentiation

10^5^ cells were seeded into 24-well plates in GM and stimulated with osteogenic media (OM) [DMEM, 10% FBS, 100 μg/mL ascorbic acid, 10 mM β-glycerophosphate, and 1% penicillin/streptomycin] the next day. Media was changed every 2 days. Alizarin Red staining (ARS) was used to visualize mineralization [32]. Quantification of ARS was performed by measuring absorbance on a Flex Station 3 microplate reader (Molecular Devices) at 405 nm in a 96-well opaque-walled transparent-bottomed plates [32]. Data were collected with Soft Max Pro (Molecular Devices) software [32]. GraphPad Prism software was used to calculate means and SEMs.

### Adipogenic differentiation

10^5^ cells SSPCs were seeded onto 24-well plates in GM. At 100% confluency, adipogenic differentiation was induced with MSC Adipogenic BulletKit (Lonza) induction/maintenance media. Media was changed every 3 days. Adipogenesis was visualized with Oil Red O staining. Quantification of staining was performed by first extracting the Oil Red O dye using 100% isopropanol and then reading absorbance on a Flex Station 3 microplate reader (Molecular Devices) at 520 nm in a 96-well opaque-walled transparent-bottomed plates [33]. Means and SEMs were calculated in GraphPad Prism software.

### Data and materials availability

Requests for data and materials should be addressed to P.L.
